# The US Supplemental Nutrition Assistance Program – Education improves nutrition-related behaviors

**DOI:** 10.1017/jns.2020.37

**Published:** 2020-09-30

**Authors:** Suzanne Ryan-Ibarra, Amy DeLisio, Heejung Bang, Omolola Adedokun, Vibha Bhargava, Karen Franck, Katie Funderburk, Jung Sun Lee, Sondra Parmer, Christopher Sneed

**Affiliations:** 1 Center for Wellness and Nutrition, Public Health Institute, 1750 Howe Ave, Suite 550, Sacramento, CA 95825, USA; 2 Public Health Sciences – Division of Biostatistics, University of California, Davis, CA 95616, USA; 3 University of Kentucky, 1500 Bull Lea Rd, Suite 130, Lexington, KY 40511, USA; 4 University of Georgia, 143 Barrow Hall, 115 DW Brooks Dr, Athens, GA 30602, USA; 5 University of Tennessee, 2621 Morgan Circle, 119 Morgan Hall, Knoxville, TN 37996, USA; 6 Alabama A&M and Auburn Universities, 205 Duncan Hall, AL 36849, USA

**Keywords:** SNAP-Ed program evaluation:, Nutrition education:, Policy, systems, and environmental changes

## Abstract

The aim of this study was to measure whether participating in Supplemental Nutrition Assistance Program – Education (SNAP-Ed) interventions is associated with changes in meeting recommendations for healthy eating and food resource management behaviours, such as shopping, among low-income children, adolescents, and adults in eight states in the US Southeast. The study used a one-group pre-test post-test design, analysing aggregate data on nutrition and shopping behaviours collected during Federal Fiscal Year 17 from SNAP-Ed direct education in community settings. Twenty-five implementing agencies in Alabama, Florida, Georgia, Kentucky, Mississippi, North Carolina, South Carolina, and Tennessee provided aggregated data on program participants. Because survey questions differed, agencies followed standard recoding guidelines. The number of participants varied depending on the indicator; the maximum number was *n* 43 303 pre-tests, *n* 43 256 post-test. Participants were significantly more likely to consume more than one kind of fruit (pooled relative risk (RR), 1⋅10; 95% confidence interval (CI), 1⋅09–1⋅11) and more than one kind of vegetable (pooled RR, 1⋅14; 95% CI, 1⋅12–1⋅15) after the intervention than before. On average, participants consumed 0⋅34 cups more of fruit per day (95% CI, 0⋅31–0⋅37), and 0⋅22 cups more of vegetables per day (95% CI, 0⋅19–0⋅25) after the intervention, compared to before. About 701 policy, systems, and environmental changes for nutrition supports were reported. This study suggests that SNAP-Ed direct education is associated with positive behaviour changes in the US Southeast. It provides a methodology that can inform data aggregation efforts across unique SNAP-Ed programs or other similar nutrition education programs to report on the collective impact.

## Introduction

The Supplemental Nutrition Assistance Program – Education (SNAP-Ed) is designed to increase the likelihood that individuals with limited budgets in the US can eat a healthy diet and achieve a physically active lifestyle, as recommended by the US Dietary Guidelines 2015–2020.^(1,2)^ SNAP-Ed is the federally funded nutrition education program of the US Supplemental Nutrition Assistance Program (SNAP). SNAP-Ed provides comprehensive nutrition education and obesity prevention interventions that target vulnerable populations (individuals and families who are at or below 185 % of the federal poverty level) across the US. State and local organisations design, implement, and evaluate diverse SNAP-Ed interventions in different locations across the US. Despite being a national program, these diverse approaches present challenges for aggregating and evaluating outcomes across agencies, states, and regions. One approach to conduct standardised SNAP-Ed evaluation culminated in the national SNAP-Ed Evaluation Framework, which provided a common set of indicators and definitions to guide evaluations of SNAP-Ed interventions.^(^^3^^)^

SNAP-Ed is administered by the United States Department of Agriculture (USDA) Food and Nutrition Service (FNS), and FNS is divided into seven geographic regions. USDA FNS provides funding to state SNAP agencies, who then provide funding to SNAP-Ed implementing agencies (IAs) to deliver SNAP-Ed interventions at the local level. IAs include universities, non-profit organisations, public health departments and faith-based organisations.

The FNS Southeast region of the US received approximately 14 % of all federal funding for SNAP-Ed in Federal Fiscal Year (FFY) 2017 ($57 549 023) and is comprised of eight US states: Alabama, Florida, Georgia, Kentucky, Mississippi, North Carolina, South Carolina and Tennessee.^(^^4^^)^ The US Southeast has some of the lowest rates of fruit and vegetable consumption and physical activity for adults and adolescents, and the highest obesity rates for adults, adolescents, and children in the nation.^(^^5^^)^ In 2017, the first regional evaluation using the national SNAP-Ed Evaluation Framework was conducted using aggregated data collected by 25 IAs in the eight states comprising the FNS US Southeast Region.

The primary aims of this study were to utilise data collected from SNAP-Ed interventions conducted during FFY 17 to (1) assess the relationship of direct nutrition education programs in the US Southeast on changes in participants’ behaviours related to healthy eating and shopping, by comparing the results of participant surveys conducted before and after the interventions, and (2) collect policy, systems, and environmental (PSE) change data related to nutrition supports.

## Methods

### Study design

**Fig. 1.**
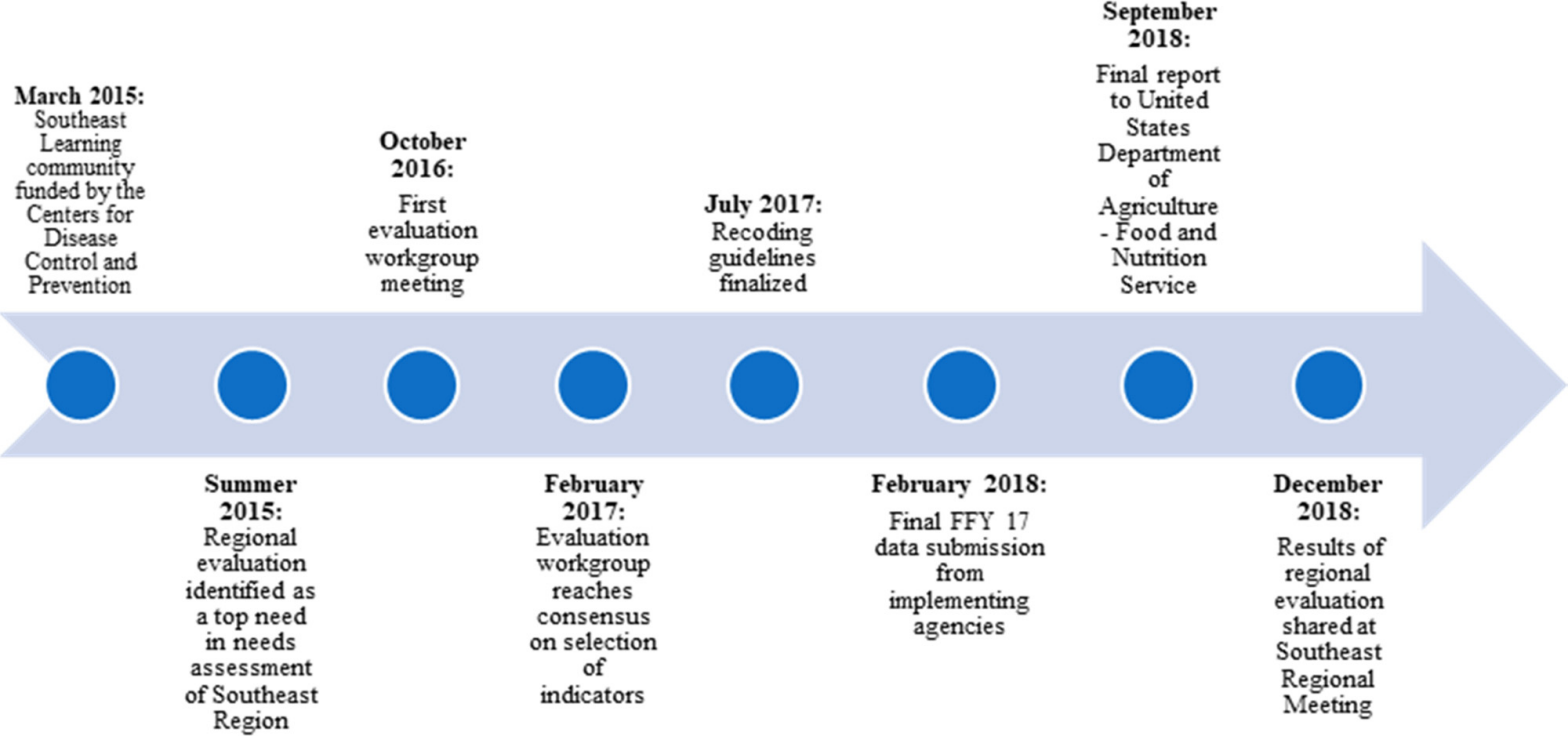
Timeline of milestones achieved in the Federal Fiscal Year 2017 US Southeast regional evaluation.

In March 2015, the Public Health Institute Center for Wellness and Nutrition (PHI CWN) facilitated a learning community to support and expand SNAP-Ed's public health and innovative strategies as funding increased in the US Southeast (Fig. 1). This learning community included many co-authors of this manuscript. The US Southeast Learning Community was a partnership between PHI CWN, USDA Southeast Regional Office (SERO), US Centers for Disease Control and Prevention, state SNAP agencies, and 25 IAs.

To prioritise regional opportunities for collaboration that would benefit the US Southeast's SNAP-Ed community, a needs assessment was conducted and a top priority identified was to collaborate on a regional evaluation of SNAP-Ed using indicators from the SNAP-Ed Evaluation Framework, which was officially released by FNS nationally on 6 June 2016 and provided a standardised method to report outcome evaluation findings from SNAP-Ed across the country.^(^^6^^)^

In October 2015, PHI CWN recruited and convened an evaluation workgroup with representatives from all eight states, including co-authors of this manuscript, to review the draft SNAP-Ed Evaluation Framework and select indicators and measures for inclusion in the regional evaluation. Twenty members consistently attended the evaluation workgroup meetings, which met virtually 1–2 times per month during FFY 2017. Through a facilitated process with the members of the regional evaluation workgroup, three indicators from the SNAP-Ed Evaluation Framework were selected to be measured: healthy eating behaviours (MT1), shopping/food resource management behaviours (MT2), and PSE for nutrition supports (MT5). US Southeast regional leadership encouraged every state to collect and report on at least one of the three selected indicators. Each IA was encouraged to the extent allowed by capacity and funding level to collect and report on the selected indicators that were hypothesised to be impacted by their current interventions. All but one state had more than one IA and were able to report on all three indicators.

### Participants

Data were provided by twenty-five SNAP-Ed IAs from all eight states in the US Southeast; representatives from all twenty-five IAs were invited to participate in this study as co-authors, and seven accepted, representing four IAs. Surveys were completed by children, teens, adults, and seniors before and after participating in interventions tailored for each age group. USDA FNS requires states and IAs to use practice-based or evidence-based curricula in their SNAP-Ed programming. Based on this existing standard, no IA was asked to modify their standard interventions or curricula because these curricula have already been deemed as effective tools to improve nutrition and food resource behaviour management among participants.^(^^6^^)^ For the analysis described here, the authors assumed that all the SNAP-Ed programming in the US Southeast was delivered as designed during FFY 17.

Each IA collected data using pre- and post-tests for healthy eating (MT1) and shopping/food resource management (MT2) behaviour indicators. The total sample size for each indicator varied because not all IAs reported on every indicator; however, the maximum sample sizes were *n* 43 303 pre-tests, and *n* 43 256 post-tests. For each SNAP-Ed program, the data were collected before (pre-test) and after (post-test) each intervention. Each IA collected evaluation data for PSE changes for nutrition supports (MT5) (*n* 701) using direct observation, interviews with key informants, repeated assessments or surveys, and photographic evidence, as recommended by the SNAP-Ed Evaluation Framework.

### Data standardisation procedures

At the time of this study, no standardised survey instrument existed to collect data on SNAP-Ed interventions. Therefore, IAs decided to use existing survey tools to collect information on individual behaviours, and the specific questions and response categories used varied by IA. To standardise responses, PHI Evaluation Team staff, led by a SNAP-Ed evaluation expert and Evaluation Framework author Sharon Sugerman, MS, RD, assessed each question and the corresponding response categories to determine whether it met the definition of meeting standards of each indicator, as described in the SNAP-Ed Evaluation Framework. For each question that met the criteria, IAs recoded responses using guidelines developed by PHI so that data could be aggregated (Supplemental Tables). Every IA entered the following summary statistics into a standardised Excel template for each of their programs: total number meeting guidelines at pre-test (for dichotomous variables) or mean and standard deviation (for continuous variables), total number who completed pre-test, total number meeting at post-test (for categorical variables) or mean and standard deviation (for continuous variables), and the total number who completed post-test.

To collect data on PSE changes, a standardised Excel template was provided that had drop-down menus for within each category (policy, systems, environmental and promotional), using the lists provided in the SNAP-Ed Evaluation Framework.^(^^3^^)^ Because the list provided was not exhaustive, IAs had the option to describe additional PSE changes in an open-ended field for PHI Evaluation Team staff to review. Reach was reported by each IA as the ‘total potential number of persons who encounter the improved environment or are affected by the change on a regular (typical) basis and are assumed to be influenced by it,’ as recommended in the SNAP-Ed Evaluation Framework.^(^^3^^)^ SNAP-Ed programs could report as many PSE changes as they wanted; however, the reach of the PSE changes at each location was counted only one time. For example, if a program reported one system change of removing sugar-sweetened beverages from children's menus with a reach of 50 children and one environmental change of improvements in layout or display of food with a reach of the same 50 children at the same school location, this was reported as implementing two PSE changes with a reach of 50 children.

### Outcome measures

Definitions for this study's outcomes were taken from the SNAP-Ed Evaluation Framework.^(^^6^^)^ Four primary outcomes related to healthy eating behaviours were analysed using the results from pre- and post-tests: (1) the percentage of participants eating more than one kind of fruit per day or week (MT1c), (2) the percentage of participants eating more than one kind of vegetable per day or week (MT1d), (3) the mean cups of fruit per day (MT1l), and (4) the mean cups of vegetables per day (MT1m). Eleven secondary outcomes related to behavioural changes were also analysed. All outcomes related to behavioural changes, including the four primary and 11 secondary outcomes, are described in Table 1. Four outcomes related to PSE changes were analysed: number of policy changes (MT5b), number of systems changes (MT5c), number of environmental changes (MT5d), and number of promotional changes to support PSE (MT5e). A sum was computed for reach (MT5f) to estimate the number of people who may have been influenced by the PSE change.

**Table 1.** Summary Healthy Eating (MT1) and Food Resource Management (MT2) behaviour changes before compared to after participating in SNAP-Ed implementing agency programs in Federal Fiscal Year 2017

Indicator	Description	Pre-test total *n*	Post-test Total *n*	Pooled RR or SMD^a^ (95 % CI)	*Q*	*I*^2^
**MT1c**	**Ate more than one kind of fruit throughout the day or week**	**39 249**	**34 838**	**1**⋅**10 (1**⋅**09–1**⋅11)*	711*	**92**⋅**8** **%**
**MT1d**	**Ate more than one kind of vegetable throughout the day or week**	**40 242**	**39 998**	**1**⋅**14 (1**⋅**12–1**⋅15)*	700*	**92**⋅**6** **%**
MT1g	Drinking water frequency	42 856	42 559	1⋅04 (1⋅03–1⋅05)*	517*	89⋅9 %
MT1h	Drinking fewer sugar-sweetened beverages	43 303	43 256	1⋅10 (1⋅09–1⋅12)*	481*	87⋅9 %
MT1i	Consuming low-fat or fat-free milk	40 533	35 698	1⋅09 (1⋅08–1⋅11)*	470*	88⋅5 %
**MT1l**^a^	**Cups of fruit consumed per day**	**8685**	**7229**	0⋅34 (0⋅31–0⋅37)*	97*	**82⋅4** **%**
**MT1m**^a^	**Cups of vegetables consumed per day**	**8684**	**7271**	0⋅22 (0⋅19–0⋅25)*	27*	**38⋅7** **%**
MT2a	Choose healthy foods for my family on a budget	15 149	11 171	1⋅40 (1⋅38–1⋅43)*	1292*	97⋅5 %
MT2b	Read nutrition facts labels or nutrition ingredients lists	16 000	12 002	1⋅66 (1⋅61–1⋅70)*	1508*	97⋅5 %
MT2g	Not run out of food before month's end	15 257	11 372	1⋅25 (1⋅22–1⋅27)*	364*	90⋅7 %
MT2h	Compare prices before buying foods	15 020	11 169	1⋅26 (1⋅23–1⋅28)*	861*	96⋅2 %
MT2i	Identify foods on sale or use coupons to save money	8770	5633	1⋅11 (1⋅08–1⋅14)*	94*	71⋅2 %
MT2j	Shop with a list	14 945	11 120	1⋅44 (1⋅41–1⋅47)*	799*	95⋅7 %

Abbreviations: CI, confidence interval; RR, relative risk; SMD, standardised mean difference

Primary outcomes are given in bold. Data presented from 25 implementing agencies.

a Standardised mean difference

* *P-*value < 0⋅05.

### Statistical analysis

IA-specific and pooled analyses were conducted to assess the relationship of direct nutrition education programs on participants’ healthy eating behaviours and food resource management skills. First, for each IA within each age group, relative risk (RR) and 95 % confidence intervals (CI) were calculated to compare the proportion of participants meeting guidelines in the pre- and post-tests for all indicators, except MT1l and MT1m. For MT1l and MT1m, the only indicators defined as continuous variables, the change in the mean cups of fruit or vegetables was analysed using *t*-tests. For IAs that provided summary data on multiple SNAP-Ed interventions, RRs were calculated for each intervention. Next, pooled RRs were conducted using meta-analyses.^(^^7^^)^ Pooled RR and 95 % CI were used to estimate the difference between the proportion of participants at all programs combined who were meeting guidelines in the post-test compared to those in the pre-test for all indicators except cups of fruit (MT1l) and vegetables (MT1m). For cups of fruit and vegetables, pooled standardised mean differences (SMDs) were used to estimate the difference in mean cups of fruit or vegetables consumed by participants in the pre-test compared to that in the post-test.

Because data were provided in the aggregate form (without unique matching identifiers or raw data), we adopted a standard meta-analytic approach for two independent groups, which would provide ‘conservative’ results.^(^^8^^)^ Random effects meta-analysis was conducted using the DerSimonian and Laird method, which estimates the pooled RR for all programs We estimated Cochran's *Q*-statistic, and *I*^2^ as heterogeneity measures; *Q* was calculated as the weighted sum of squared differences between each IA's results and the pooled effect across all IAs, and *I*^2^ was calculated as the percentage of variation across studies that is due to heterogeneity, not chance.^(^^9^^)^ We used random effect models because we assumed that effect sizes vary across IAs, not only because of sampling errors but also because of differences specific to the unique context of each IA, including factors such as audience type, location, educators’ effectiveness, and number of classes in each curriculum.^(^^10^^)^

To examine if results differed by age, subgroup analyses were conducted by age group: children (0–11 years), teens (12–17 years), adults (18–59 years), and seniors (60 years or older).

Descriptive analyses of PSE changes were performed. Counts were computed for all PSE types (policy, systems, and environmental) as well as promotional efforts for PSE. A sum was computed for reach. The total number of PSE changes taking place in each PSE setting (e.g., worksites, schools, and food stores) were also counted.

All analyses were conducted using SAS version 9.4 (Cary, NC) or Stata version 10.1 (College Station, TX). Two PHI researchers independently conducted analyses using identical methodologies and verified their results were identical. Our analyses were not adjusted for multiple comparisons.

This study was considered exempt by the Public Health Institute's Institutional Review Board, #I17-012.

## Results

Data were received from SNAP-Ed programs reaching children (0–11 years), teens (12–17 years), adults (18–59 years), and seniors (60 years or older). Most of the data received was from programs that surveyed adults (38 %) or children (36 %), with smaller percentages of data received from programs that surveyed seniors (14 %) or teens (12%).

Results from pre- and post-tests show that participants in SNAP-Ed programs are statistically significantly more likely to meet the recommendations from the Dietary Guidelines for Americans for healthy eating behaviours and have positive food resource management behaviours after participating in the programs, compared to before (Table 1).

SNAP-Ed in the US Southeast was associated with significant improvements in fruit and vegetable consumption, the primary outcome of this study. Participants in SNAP-Ed programs were more likely to consume more than one kind of fruit (pooled RR, 1⋅10; 95 % CI, 1⋅09–1⋅11) and more than one kind of vegetable (pooled RR, 1⋅14; 95 % CI, 1⋅12–1⋅15) after the intervention than before. On average, cups of fruit per day increased by 0⋅34 cups (SMD = 0⋅36; 95 % CI, 0⋅31–0⋅37), and cups of vegetables per day increased by 0⋅22 cups (SMD = 0⋅22; 95 % CI, 0⋅19–0⋅25). Findings were similar for children, teens, adults, and seniors in subgroup analyses by age, except cups of fruit per day did not significantly increase for children and cups of vegetables per day did not significantly increase for teens (Table 2). Findings were significant and positive for the following secondary outcomes for healthy eating behaviours: drinking water frequently (pooled RR, 1⋅04; 95 % CI, 1⋅03–1⋅05), drinking fewer sugar-sweetened beverages (pooled RR, 1⋅10; 95 % CI, 1⋅09–1⋅12), and consuming low-fat and fat-free milk (pooled RR, 1⋅09; 95 % CI, 1⋅08–1⋅11).

**Table 2.** Summary Healthy Eating (MT1) and Shopping/Food Resource Management (MT2) behaviour changes before compared to after participating in SNAP-Ed implementing agency programs in Federal Fiscal Year 2017, compared by age group

Indicator	Description	Children (2–11 years)^a^	Teens (12–17 years)	Adults (18–59 years)	Seniors (60+ years)		
		Pooled RR or SMD^b^ (95 % CI)				Q	I^2^
**MT1c**	**Ate more than one kind of fruit throughout the day or week**	1⋅04* (1⋅03–1⋅06)	1⋅08* (1⋅05–1⋅11)	1⋅48* (1⋅43–1⋅54)	1⋅15* (1⋅10–1⋅19)	711*	**92⋅8** **%**
**MT1d**	**Ate more than one kind of vegetable throughout the day or week**	1⋅09* (1⋅07–1⋅11)	1⋅18* (1⋅13–1⋅24)	1⋅30* (1⋅26–1⋅34)	1⋅11* (1⋅07–1⋅14)	700*	**92⋅6** **%**
MT1g	Drinking water frequency	1⋅02* (1⋅01–1⋅03)	1⋅01 (0⋅996–1⋅03)	1⋅18* (1⋅15–1⋅20)	1⋅03* (1⋅009–1⋅06)	517*	89⋅9 %
MT1h	Drinking fewer sugar-sweetened beverages	1⋅08* (1⋅06–1⋅10)	1⋅07* (1⋅03–1⋅11)	1⋅24* (1⋅20–1⋅29)	1⋅11* (1⋅06–1⋅16)	481*	87⋅9 %
**MT1i**	**Consuming low-fat or fat-free milk**	1⋅07* (1⋅05–1⋅09)	1⋅01 (0⋅98–1⋅04)	1⋅33* (1⋅29–1⋅38)	1⋅09* (1⋅04–1⋅13)	470*	88⋅5 %
**MT1l**^b^	**Cups of fruit consumed per day**	0⋅07 (−0⋅06–0⋅19)	0⋅32* (0⋅11–0⋅54)	0⋅36* (0⋅33–0⋅40)	0⋅33* (0⋅25–0⋅42)	97*	**82⋅4** **%**
MT1mb^b^	Cups of vegetables consumed per day	0⋅15* (0⋅03–0⋅28)	0⋅21 (−0⋅01–0⋅42)	0⋅22* (0⋅18–0⋅25)	0⋅27* (0⋅18–0⋅35)	27*	**38⋅7** **%**
MT2a	Choose healthy foods for my family on a budget			1⋅49* (1⋅45–1⋅52)	1⋅17* (1⋅13–1⋅21)	1292*	97⋅5 %
MT2b	Read nutrition facts labels or nutrition ingredients lists		2⋅61* (1⋅59–4⋅28)	1⋅91* (1⋅84–1⋅98)	1⋅19* (1⋅14–1⋅24)	1508*	97⋅5 %
MT2g	Not run out of food before month's end			1⋅32* (1⋅29–1⋅35)	0⋅95 (0⋅90–1⋅0001)	364*	90⋅7 %
MT2h	Compare prices before buying foods			1⋅33* (1⋅30–1⋅35)	1⋅07* (1⋅03–1⋅11)	861*	96⋅2 %
MT2i	Identify foods on sale or use coupons to save money		0⋅93 (0⋅75–1⋅15)	1⋅12* (1⋅08–1⋅17)	1⋅11* (1⋅06–1⋅15)	94*	71⋅2 %
MT2j	Shop with a list			1⋅54* (1⋅50–1⋅58)	1⋅16* (1⋅11–1⋅21)	799*	95⋅7 %

Abbreviations: CI, confidence interval; RR, relative risk; SMD, standardised mean difference

Primary outcomes are given in bold⋅

a Data for children not reported for MT2 indicators due to small sample size for reporting (two IAs)⋅

b Standardised mean difference⋅

* *P-*value < 0⋅05.

Findings were significant and positive for the following secondary outcomes for shopping/food resource management behaviours. Specifically, participants were more likely to choose healthy foods for their family on a budget (pooled RR, 1⋅44; 95 % CI, 1⋅38–1⋅43), read nutrition facts labels (pooled RR, 1⋅66; 95 % CI, 1⋅61–1⋅71), have increased food security (as defined by not running out of food before month's end; pooled RR, 1⋅25; 95 % CI, 1⋅22–1⋅27), compare prices before buying foods (pooled RR, 1⋅26; 95 % CI, 1⋅23–1⋅28), identify foods on sale (pooled RR, 1⋅11; 95 % CI, 1⋅08–1⋅14), and shop with a list after participating in the program (pooled RR, 1⋅44; 95 % CI, 1⋅41–1⋅47), compared to that before.

A total of 701 PSE changes reached 830 049 people (Table 3). The highest number of PSE changes were environmental changes (*n* 357), followed by systems changes (*n* 245), and policy changes (*n* 99). These PSE changes were supported by 471 promotional efforts. The highest number of PSE changes was reported in the learn domain (*n* 580), defined in the SNAP-Ed Evaluation Framework as changes that occurred in schools, before- and after-school programs, early care and education facilities, libraries and other places where people go to learn, and the lowest number of PSE changes was reported in the work domain (*n* 4), defined in the SNAP-Ed Evaluation Framework as changes that occurred in adult education, job training, worksite, and other places where people go to work. The most common environmental changes reported were edible gardens (*n* 242) and improvements in layout or display of food, such as in school cafeterias (*n* 33). The most common system changes reported were prioritising farm to table or an increase in fresh or local produce (*n* 61) and improving child feeding practices (*n* 28). The most common policy changes reported were implementing standards for healthier food policies (*n* 32) and school wellness or childcare wellness policies (*n* 17).

**Table 3.** Counts of policy, systems, environmental (PSE), and promotional changes adopted in the Federal Fiscal Year 2017 in the US Southeast

	Total nutrition supports adopted (MT5b + MT5c + MT5d)	Reach (MT5f)^a^	Policy changes (MT5b)	Systems changes (MT5c)	Environmental changes (MT5d)	Promotional changes for PSE (MT5e)
Total	701	830 049	99	245	357	471
Domain^b^						
Eat	35	17 377	3	7	25	31
Learn	396	316 679	55	119	222	200
Live	45	4068	5	10	30	88
Play	22	20 918	1	9	12	14
Shop	158	457 159	28	80	50	98
Work	4	13 555	0	0	4	25

Abbreviation: IA, implementing agency

a Reach counts do not include reach reported for promotional efforts (MT5e) only

b Totals for domains may not add to PSE change totals in each row. This is because some programs selected multiple MT5 nutrition support types for a PSE intervention, but these interventions were only counted once in the domains.

## Discussion

This study established a methodology to aggregate quantitative data from 25 SNAP-Ed IAs across the FNS's US Southeast region. Using this methodology, our study found that SNAP-Ed programs in the US Southeast are associated with significant improvements in self-reported healthy eating behaviours and food resource management behaviours. These significant improvements were observed in the overall sample, as well as in subgroup analyses by age group. In addition, we found that the direct education offered through SNAP-Ed programs in this region is complemented by PSE changes, as well as marketing and promotion designed to increase awareness of the PSE changes. Residents of the US Southeast experience some of the highest rates of obesity and unhealthy dietary behaviours in the nation. Our study shows that SNAP-Ed programs can be part of the solution to improve dietary behaviours and nutrition environments in the region.

The IAs in our study have published previously that their efforts are associated with statistically significant improvements in eating behaviours.^(11,12)^ However, to our knowledge, ours is the first study to aggregate data from unique SNAP-Ed programs to examine the combined outcomes of these programs at the regional level. This approach can indicate the potential impact of these programs in the region. Furthermore, it provides evidence to suggest that SNAP-Ed programs are effective and the investment at the federal level has merit.

SNAP-Ed programs are designed to approach obesity prevention using a comprehensive approach based on the Social Ecological Model.^(^^3^^)^ The focus is to provide direct education and skill-based classes to influence individual and interpersonal behaviours in community settings as the first two layers of the model and then to implement PSE changes in organisation and community settings and ultimately influence policy and norms to make the healthy choice, the easy choice. SNAP-Ed also uses social marketing via billboards, social media, and other marketing and promotional materials with health education messages to influence these societal changes.^(^^6^^)^ Our study measures two main components of SNAP-Ed's comprehensive approach: direct nutrition education and PSE for nutrition supports.

To ensure comparability of the data collected by numerous IAs across the region, our study methods followed procedures to develop a standard set of guidelines for recoding of responses. To ease the burden on IAs, we collected summary statistics instead of individual-level data; studies suggest efficiency lost could be minimal with summary statistics, as in standard meta-analysis.^(^^13^^)^ Therefore, we believe that our analysis benefits from a methodology that has been documented as a rigorous approach to calculate pooled effect estimates and may demonstrate that summary statistics from aggregate data can serve as efficient information gathering toward valid analyses in similar settings (e.g., data monitoring, evaluation, and surveillance), although individual participant data are always in a gold standard.^(^^13^^)^

However, bias may be introduced because individual-level covariates were not adjusted for in the summary statistics provided for this analysis.^(^^4^^)^ In separate, unpublished analyses from the US state of Georgia using individual-level data conducted by the authors, we found differences in subgroup analyses by gender and race/ethnicity for some indicators, which suggests that similar differences could exist in the data from other states in the US Southeast as well. Future analyses should adjust for individual-level covariates that are potential confounders.

Strengths of our study include that, to our knowledge, it is the largest quantitative multi-state evaluation of SNAP-Ed programs to date that analysed data submitted by twenty-five implementing agencies. Furthermore, we used a standard meta-analytic approach to conduct statistical testing that permitted a quantitative, regional evaluation of outcomes related to SNAP-Ed. SNAP-Ed programs use diverse curricula that allow them to tailor their approach to the varied contexts of each community.^(^^6^^)^ Furthermore, these programs measure program outcomes using different, yet validated, survey instruments.^(^^6^^)^ Therefore, it has been methodologically challenging to obtain an overall estimate for the regional outcomes related to SNAP-Ed programs. However, using a standard meta-analytic approach allows us to compare the results of each IA's SNAP-Ed program, with participants grouped together, yet also obtain summary statistics of the weighted average of SNAP-Ed outcomes across the US Southeast.

This study had limitations related to the individual-behaviour data and the PSE data. First, this study used self-reported data from pre- and post-tests to measure healthy eating and food resource management behaviours, without objective outcomes or further validation. Due to the data being self-reported, there may be some inaccuracy in reporting due to social desirability bias. Furthermore, we did not receive individual-level data so we could not assess whether the demographics of participants who completed both pre- and post-tests was different from that of participants who did not complete the post-test.

Second, there was no independent control group, so this study cannot assess whether behaviour change would have been observed in the absence of the SNAP-Ed programming. We also did not have access to unique identification numbers linking pre- to post-test responses, so we could not conduct a matched/paired pre–post analysis. However, we used a conservative variance formula.

Third, some of the instruments used to collect data on healthy eating behaviours and food resource management behaviours used response categories that allowed for more detailed responses than this study's recoding guidelines, which were dichotomous (e.g. Likert-type scales, food frequency and rating). An unpublished sensitivity analysis conducted using data from the US state of Alabama showed that positive improvements were detected as statistically significant using the original response categories but not the categories as recoded into a dichotomous variable, using the guidelines from our study. This suggests that our analysis was less sensitive (e.g., information loss) to detecting change than it could have been.^(^^14^^)^ This loss of statistical power was expected as an artefact of recoding the outcomes of interest as dichotomous for standardisation. Ideally, data collection would be conducted using identical instruments so that recoding would be unnecessary. Other possible methodological approaches include calculating mean scores for each IA, maintaining the original response categories and standardising to compare across IAs using effect size estimates. However, the current methods allowed us to successfully combine data from 25 IAs, despite using different instruments, to measure the outcomes of SNAP-Ed programs conducted across the US Southeast. Furthermore, the recoding strategy we applied allows the communication of the results in a straightforward way as two-category variables with yes/no responses or as mean cups of fruits or vegetables; similar methods have been used in other US government research.^(^^15^^)^ Although by recoding the response categories from multiple instruments and by not having access to the raw data, we were not able to verify that the original validity of the instruments was retained and should be considered in future studies.

Fourth, the set of data provided, and the analyses of those data, are limited to PSE changes that have already been implemented and documented. IAs were asked to identify, for each PSE reported, the method of documentation they used to confirm the implementation. Despite this and other precautions to ensure data quality, there is some subjectivity inherent in the reporting of PSEs. Therefore, IAs may not have accurately reported the reach since the estimation guidelines are more subjective in some community-based settings (e.g. famers market) than others (e.g. schools), who have access to more precise numbers of people impacted.

## Conclusions

SNAP-Ed interventions in the US Southeast are associated with significant improvements in self-reported healthy eating and shopping/food resource management behaviours among adult, teen, and child participants. Furthermore, the improvements in individual behaviours are complemented by PSE changes throughout the region that reached more than three-quarters of a million low-income residents.

Because the focus of this study is to present the results of the regional evaluation, we did not include the results of state-specific analyses. These efforts in direct education and PSE changes are taking place in every state throughout the US Southeast; however, our study found that the magnitude of the findings for individual-behaviour change is not equal in every state. Our analysis detected a stronger impact of SNAP-Ed programs in some states compared to others; in addition, our analysis detected that some SNAP-Ed programs led by IAs had stronger impacts on healthy eating and food resource management behaviours than others. This provides an opportunity to offer targeted technical assistance to those states and IAs whose efforts can be strengthened to improve the health of their individual participants. It also can allow for further exploration of the programs in states with stronger results to review for scalability of these approaches.

This study provides evidence that SNAP-Ed programs using a comprehensive approach of education and PSE changes provide opportunities for low-income people in the US Southeast to adopt healthier behaviours, which may reduce the burden of diet-related disease and healthcare costs in the region. Furthermore, it may influence additional federal and state funding to be dedicated to nutrition programs that can positively impact the health of Americans living in poverty.

The methodology developed and utilised in this study can inform data aggregation efforts of SNAP-Ed and similar prevention programs to report collective outcomes in other FNS regions. This methodology may be considered as one way to conduct state, regional, and national evaluations and provide support for a national approach to evaluation and technical assistance for SNAP-Ed IAs, as required in the Agriculture and Improvement Act of 2018, also known as the Farm Bill.^(^^16^^)^

## Supplementary material

For supplementary material accompanying this paper visit https://doi.org/10.1017/jns.2020.37. click here to view supplementary material
