# Supplementary material for: The US Supplemental Nutrition Assistance Program – Education improves nutrition-related behaviors
Source: J Nutr Sci. 2020 Sep 30;9:e44. doi: 10.1017/jns.2020.37 (PMC7731638; doi:10.1017/jns.2020.37)
Supplement: Supplementary file 1 [file S2048679020000373sup001.docx]

**SUPPLEMENTAL MATERIALS**

[Supplemental Table 1. Instrument recoding guidelines for eat more than one kind of fruit (MT1c) 2](#_Toc36130010)

[Supplemental Table 2. Instrument recoding guidelines for eat more than one kind of vegetable (MT1d) 3](#_Toc36130011)

[Supplemental Table 3. Instrument recoding guidelines for drinking water frequently (MT1g) 5](#_Toc36130012)

[Supplemental Table 4. Instrument recoding guidelines for drinking fewer sugar-sweetened beverages (MT1h) 7](#_Toc36130013)

[Supplemental Table 5. Instrument recoding guidelines for low-fat or fat-free milk (MT1i) 11](#_Toc36130014)

[Supplemental Table 6. Instrument recoding guidelines for cups of fruit (MT1l) 13](#_Toc36130015)

[Supplemental Table 7. Instrument recoding guidelines for cups of vegetables (MT1m) 14](#_Toc36130016)

[Supplemental Table 8. Instrument recoding guidelines for choose healthy foods for my family on a budget (MT2a) 15](#_Toc36130017)

[Supplemental Table 9. Instrument recoding guidelines for read nutrition facts labels or ingredients lists (MT2b) 16](#_Toc36130018)

[Supplemental Table 10. Instrument recoding guidelines for not run out of food before month’s end (MT2g) 18](#_Toc36130019)

[Supplemental Table 11. Instrument recoding guidelines for compare prices before buying foods (MT2h) 20](#_Toc36130020)

[Supplemental Table 12. Instrument recoding guidelines for identify foods on sale or use coupons to save money (MT2i) 22](#_Toc36130021)

[Supplemental Table 13. Instrument recoding guidelines for shop with a list (MT2j) 23](#_Toc36130022)

[Supplemental Table 14. Categories Used to Report Policy, Systems, Environmental, and Promotional Changes (MT5)^a^ 25](#_Toc36130023)

Supplemental Table 1. Instrument recoding guidelines for eat more than one kind of fruit (MT1c)

| **Question**  ***Source(s)*** | **Response Categories** | **Recode of Response Categories** |
| --- | --- | --- |
| 24-hour recall | Cups, open-ended text | 1 = Yes, ate more than 1 kind of fruit during 24-hour recall  0 = No, did not eat more than 1 kind of fruit during 24-hour recall |
| Do you eat more than one kind of fruit each day? (with image)  *Body Quest Parent Survey* | No Yes, sometimes Yes, often Yes, always | 1 = Yes, always/Yes, often 0 = Yes, sometimes/No |
| Do you eat more than one kind of fruit each day?  *WebNEERS* | N/A(=0)  Never (=1)  Seldom(=2)  Sometimes(=3)  Most times (=4)  Always (=5) | 1 = Always/Most times 0 = Sometimes/Seldom/Never/NA |
| Do you eat more than one kind of fruit each day?  *Food Stamp Food Behavior Checklist* | No Yes, sometimes Yes, often Yes, always | 1 = Yes, always/Yes, often 0 = Yes, sometimes/No |
| I eat more than one kind of fruit.  *Youth Behavior Survey, Grades 3-5 and 6-12*  *Adult Behavior Survey* | 0 days  1-3 days  4-6 days  7 days | 1 = 4-6 days/7 days 0 = 0 days/1-3 days |
| I (and the people in my home) eat more than one kind of fruit.  *Recipe for Success Survey* | Never Seldom Sometimes Most times Always | 1 = Always/Often 0 = Sometimes/Rarely/Never |
| My child now eats more than one kind of fruit each day.  *Parent Survey, Alabama Department of Public Health* | Yes  No | 1 = Yes  0 = No |
| I eat more than one kind of fruit.  *Homestyles* | 0 days  1-3 days 4-6 days  7 days | 1 = 7 days/4-6 days 0 = 1-3 days/0 days |
| My child eats more than one kind of fruit.  *MyPlate for my Family* | 0 days  1-3 days 4-6 days  7 days | 1 = 7 days/4-6 days 0 = 1-3 days/0 days |
| My child now eats more than one kind of fruit each day.  *Alabama Department of Public Health Parent Survey* | Never  Some days  Most days  Every day. | 1 = Every day/Most days 0 = Some days/Never |

Supplemental Table 2. Instrument recoding guidelines for eat more than one kind of vegetable (MT1d)

| **Question**  ***Source(s)*** | **Response Categories** | **Recode of Response Categories** |
| --- | --- | --- |
| 24-hour recall | Cups, open-ended text | 1 = Yes, ate more than 1 kind of vegetable during 24-hour recall  0 = No, did not eat more than 1 kind of vegetable during 24-hour recall |
| Do you eat more than one kind of vegetable each day?  *WebNEERS* | N/A Never  Seldom  Sometimes  Most times  Always | 1 = Always/Most times 0 = Sometimes/Seldom/Never/NA |
| Do you eat more than one kind of vegetable each day?  *Food Stamp Food Behavior Checklist* | No Yes, sometimes Yes, often Yes, always | 1 = Yes, always/Yes, often 0 = Yes, sometimes/No |
| How often do you eat more than one kind of vegetable a day?  *Live Well Alabama* | Never  Rarely  Sometimes  Often  Always | 1 = Always/Often 0 = Sometimes/Rarely/Never |
| How often do you eat more than one KIND of vegetable?  *POC (Youth), Steps for Health, North Carolina State University* | Never  Sometimes  Always | 1 = Always 0 = Sometimes/Never |
| I eat more than one kind of vegetable.  *Youth Behavior Survey, Grades 3-5* | Never  Some days  Most days  Every day | 1 = Every day/Most days 0 = Some days/Never |
| I eat more than one kind of vegetable.  *Youth Behavior Survey, Grades 6-12*  *Adult Behavior Survey* | 0 days  1-3 days  4-6 days  7 days | 1 = 7 days/4-6 days 0 = 1-3 days/0 days |
| My child now eats more than one kind of vegetable eat day.  *Alabama Department of Public Health Parent Survey* | No  Yes | 1 = Yes 0 = No |
| I eat more than one kind of vegetable.  *Homestyles* | 0 days  1-3 days  4-6 days  7 days | 1 = 7 days/4-6 days 0 = 1-3 days/0 days |
| My child eats more than one kind of vegetable.  *MyPlate for my Family* | 0 days  1-3 days  4-6 days  7 days | 1 = 7 days/4-6 days 0 = 1-3 days/0 days |
| I (and the people in my home) eat more than one kind of vegetable.  *Recipe for Success Survey* | Never  Seldom  Sometimes  Most times  Always | 1 = Always/Most times 0 = Sometimes/Seldom/Never |
| How often do you eat more than one KIND of vegetable?  *WEALTH - Adult* | Never  Seldom  Sometimes  Most of the time  Always | 1 = Always/Most of the time 0 = Sometimes/Seldom/Never |

Supplemental Table 3. Instrument recoding guidelines for drinking water frequently (MT1g)

| **Question**  ***Source(s)*** | **Response Categories** | **Recode of Response Categories** |
| --- | --- | --- |
| Yesterday, did you drink any water, such as from a bottle, a glass, or a water fountain?  *Body Quest* | No, I did not drink any water yesterday.  Yes, I drank water 1 time yesterday.  Yes, I drank water 2 times yesterday.  Yes, I drank water 3 times yesterday.  Yes, I drank water 4 times yesterday.  Yes, I drank water 5 or more times yesterday. | 1 = Yes, I drank water 1-5 or more times yesterday. 0 = No, I did not drink water yesterday. |
| How often do you typically drink a bottle or a glass of water? (Count tap, bottled and sparkling water).  *Body Quest Parent* | Not at all Once a week or less More than once a week Once a day More than once a day | 1 = More than once a day/Once a day 0 = More than once a week/Once a week or less/Not at all |
| How often did you drink these beverages in the past week? Unflavored bottled water, tap water, water from a drinking fountain or other unflavored water  *Beverage Screener Questionnaire* | Never or less than 1 per week  1 per week  2-4 per week  5-6 per week  1 per day  2-3 per day 4+ per day | 1 = 1-4+ per day 0 = 5-1 per week/Never or less than 1 per week |
| How often do you typically drink a bottle or glass of water? (Count tap, bottled and sparkling water.)  *Cooking Matters (adult, teen)* | Not at all  Once a week or less  More than once a week  Once a day  More than once a day | 1 = More than once a day/once a day 0 = More than once a week/Once a week or less/Not at all |
| How often do you drink water (count tap, bottled, or sparking water)?  *EFNEP* | Never  Once in a while  Once each day  Twice each day  3 or more times a day | 1 = 3 or more times a day/Twice each day 0 = Once/Once in a while/Never |
| How often do you typically drink a bottle or glass of water? *WebNEERS* | Don't do  1-2 times a day  3-4 times a day  5 times or more a day | 1 = 1-5 times or more a day 0 = Don't do |
| I (and the people in my home) drink plain water.  *Recipe for Success Survey* | Never  Seldom  Sometimes  Most times  Always | 1 = Always/Most times 0 = Sometimes/Seldom/Never |
| Yesterday, did you drink any water, such as from a glass, a bottle, or a water fountain?  *Youth Nutrition and Physical Activity Questionnaire* | No  Yes 1 time yesterday  Yes 2 times yesterday  Yes 3 times yesterday  Yes 4 times yesterday  Yes 5 or more times yesterday | 1 = 1-5 or more times yesterday 0 = No |
| How often do typically…drink water?  *University of South Carolina* | Not at all  Once a week or less  More than once a week  Once a day  More than once a day | 1 = More than once a day/Once a day 0 = More than once a week/Once a week or less/Not at all |
| How often do you drink plain water?  *POC (Youth), Steps for Health, North Carolina State University* | Never  Sometimes  Always | 1 = Always 0 = Sometimes/Never |
| How often do you drink plain water?  *Alabama Department of Public Health – adults and youth* | None  2 or 3 times per week  Every day | 1 = Every day 0 = 2 or 3 times a week/None |
| I drink plain water.  *Homestyles*  *MyPlate for my Family* | 0 days 1-3 days 4-6 days 7 days | 1 = 7 days/4-6 days 0 = 1-3 days/0 days |
| How often do you drink more plain water?  *WEALTH (adult)* | Nevers  Seldom  Sometimes  Most of the time  Always | 1 = Always/Most of the time 0 = Sometimes/Seldom/Never |
| I drink plain water.  *Youth Behavior Survey, Grades 6-12*  *Adult Behavior Survey* | 0 days  1-3 days  4-6 days  7 days. | 1 = 7 days/4-6 days 0 = 1-3 days/0 days |

Supplemental Table 4. Instrument recoding guidelines for drinking fewer sugar-sweetened beverages (MT1h)

| **Question**  ***Source(s)*** | **Response Categories** | **Recode of Response Categories** |
| --- | --- | --- |
| 1: Yesterday, did you drink any punch, sports drinks, or other fruit-flavored drinks? Do not count 100% fruit juice or diet drinks.  2: Yesterday, did you drink any regular (not diet) sodas or soft drinks?  *Body Quest* | 1: No, I did not drink any of these drinks yesterday.  Yes, I drank one of these drinks 1 time yesterday.  Yes, I drank one of these drinks 2 times yesterday.  Yes, I drank one of these drinks 3 times yesterday. Yes, I drank one of these drinks 4 times yesterday.  Yes, I drank one of these drinks 5 or more times yesterday.   2: No, I did not drink any of these regular (not diet) sodas or soft drinks yesterday.  Yes, I drank regular (not diet) sodas or soft drinks 1 time yesterday.  Yes, I drank regular (not diet) sodas or soft drinks 2 times yesterday.  Yes, I drank regular (not diet) sodas or soft drinks 3 times yesterday.  Yes, I drank regular (not diet) sodas or soft drinks 4 times yesterday. Yes, I drank regular (not diet) sodas or soft drinks 5 or more times yesterday. | 1 = No 0 = Yes, I drank one of these drinks yesterday…5 or more times yesterday  OR  Yes, I drank regular (not diet) sodas yesterday…5 or more times yesterday. |
| How often do you drink:   - Fruit drinks (such as Snapple, flavored teas, Capri Sun and Kool-Aid) - Sport drinks (such as Gatorade or PowerAde); these drinks usually do not have caffeine - Flavored waters such as Propel or vitamin waters; these drinks usually do not have caffeine - Diet soda or pop (include all kinds such as Diet Pepsi, Pepsi One, Diet Coke, Diet 7-Up) - Regular soda or pop (include all kinds such as Coke, Pepsi, 7-Up, Sprite, root beer) - Energy drinks (such as Rockstar, Red Bull, Monster and Full Throttle);   How often do you typically drink:   - 100% fruit juices like orange juice, apple juice or grape juice? (Do not count punch, Kool-aid, sports drinks or other fruit-flavored drinks.) - A can, bottle, or glass of regular soda or pop, sports drink, or energy drink? (Do not count diet or zero calorie drinks.)   *Beverage Screener Questionnaire* | Never or less than 1 per week  1 per week  2-4 per week  5-6 per week  1 per day  2-3 per day 4+ per day | 1 = Sum of times drinking: Fruit drinks, Sports drinks, Flavored waters, Regular soda, and Energy drinks is Never/Less than 1 per week/1 per week 0 = Sum of times drinking: Fruit drinks, Sports drinks, Flavored water, Regular soda, and Energy drinks is 2-6 per week/1-4+ per day |
| How often do you typically drink a can, bottle, or glass of regular soda or pop, sports drink, or energy drink? (Do not count diet or zero calorie drinks.) *Cooking Matters (adult, teen)* | Not at all  Once a week or less  More than once a week  Once a day  More than once a day | 1 = Not at all/Once a week or less 0 = More than once a week/Once a day/More than once a day |
| Yesterday, how many times did you drink sweetened drinks like soda, fruit-flavored drinks, sports drinks, energy drinks and vitamin water? Do not include 100% fruit juice.  How often do you drink regular (not diet) soda?  *EFNEP* | None  1 time  2 times  3 times  4 or more times | 1 = None 0 = 1-4 or more times |
| How often do you drink regular (not diet) soda?  How often do you drink sugar-sweetened beverages?  *WebNEERS* | Don't do  Seldom  Sometimes  Most of the time  Almost always | 1 = Don't do 0 = Seldom/Sometimes/Most of the time/Almost always |
| During the past 7 days, how many times did you drink a can, bottle, or glass of soda, such as Coke, Pepsi or Sprite? (Do not count diet soda)  *HealthMPowers* | I never drink soda I did not drink soda during the past 7 days 1 to 3 times during past 7 days 4 to 6 times during past 7 days 1 time per day; 2 times per day 3 times per day 4 or more times per day | 1 = I never drink soda/I did not drink soda during the past 7 days 0 = 1-6 times during the past 7 days/1-4 or more times per day |
| Yesterday, did you drink any punch, sports drinks, or other fruit flavored drinks? (Do not count 100% fruit juice or diet drinks)  *Mississippi State University Extension Service* | No, I didn't drink any of these drinks yesterday.  Yes, I drank one of these drinks 1 time yesterday.  Yes, I drank one of these drinks 2 times yesterday.  Yes, I drank one of these drinks 3 or more times yesterday. | 1 = No, I didn't drink any of these drinks yesterday. 0 = Yes, I drank one of these drinks 1-3 or more times yesterday. |
| How often do you typically drink ... regular soda?  *University of South Carolina* | Not at all  Once a week or less  More than once a week  Once a day  More than once a day | 1 = Not at all/Once a week or less 0 = More than once a week/Once a day/More than once day |
| How often do you drink sugary beverages?  *POC (Youth), Steps for Health, North Carolina State University* | Never  Sometimes  Always | 1 = Never 0 = Sometimes/Always |
| Yesterday, did you drink any punch, sports drinks or other fruit-flavored drinks? Do not count 100% fruit juice or diet drinks.  *Youth Nutrition and Physical Activity Questionnaire* | No, I didn’t drink any punch, sports drinks, or other fruit-flavored drinks yesterday. Yes, I drank punch, sports drinks, or other fruit-flavored drinks 1 time yesterday.  Yes, I drank punch, sports drinks, or other fruit-flavored drinks 2 times yesterday.  Yes, I drank punch, sports drinks, or other fruit-flavored drinks 3 or more times yesterday. | 1 = No, I didn't drink any punch, sports drinks, or other fruit-flavored drinks (not diet, not 100% juice) yesterday. 0 = Yes, I drank punch, sports drinks, or other fruit-flavored drinks (not diet, not 100% juice) 1-3 or more times yesterday. |
| How often do you drink soda or tea?  *Alabama Department of Public Health (adults and youth)* | None  2 or 3 times a week  Every day | 1 = None 0 = 2 or 3 times a week/Every day |
| I drink sugary beverages (like soda, fruit, drinks, or sports drinks).  *Youth Behavior Survey, Grades 3-5, 6-12* | Never  Some days  Most days  Every day | 1 = Never 0 = Some days/Most days/Every day |
| I drink sugary beverages (like soda, fruit, drinks, or sports drinks).  *Adult Behavior Survey* | 0 days  1-3 days  4-6 days  7 days | 1 = 0 days 0 = 1-7 days |
| Yesterday, did you drink any regular (not diet) sodas or soft drinks?  *Auburn University* | No I didn't drink any regular (not diet) sodas or soft drinks  Yes 1 time  Yes 2 times  Yes 3 or more times | 1 = No, I didn't drink any regular sodas or soft drinks 0 = Yes, 1-3 or more times |
| How often do you typically drink any punch, Kool-Aid, sports drink, fruit flavored drinks?" and "How often do you typically drink any regular soft drinks?  *University of South Carolina* | Not at all  Once a week  More than once a week  Once a day  More than once a day | 1 = Not at all/Once a week 0 = More than once a week/Once a day/More than once a day |
| How often do you drink fewer sugary beverages?  *WEALTH (adult)* | Always  Most of the time  Sometimes  Seldom  Never | 1= Never/Seldom 0 = Sometimes/Most of the time/Always |
| I drink sugary beverages (like soda, fruit, drinks, or sports drinks).  *Homestyles*  *MyPlate for my Family* | 0 days  1-3 days  4-6 days  7 days | 1 = 0 days 0 = 1-7 days |
| How often do you drink regular (not diet) soda, sweet tea, sugar-sweetened fruit drinks (such as lemonade), sports drinks or punch?  *University of Georgia* | Almost never 1-3 days each week 4-6 days each week Once each day 2 or more times each day | 1 = Almost never 0 = 1-3 days each week/4-6 days each week/Once each day/2 or more times each day |

Supplemental Table 5. Instrument recoding guidelines for low-fat or fat-free milk (MT1i)

| **Question**  ***Source(s)*** | **Response Categories** | **Recode of Response Categories** |
| --- | --- | --- |
| What type of milk do you drink most of the time? Choose only one.  *Body Quest* | Regular (whole) milk  2% milk  1% (low-fat) or fat-free (skim/non-fat) milk  Soy milk, almond milk, rice milk, or other milk  I do not drink milk.  I do not know. | 1 = 1% (low-fat) or fat-free (skim/non-fat) milk 0 = Regular (whole) milk/2% milk [blank] = soy milk, almond milk, rice milk, or other milk/I do not drink milk/I do not know. |
| How often do you drink 1% or nonfat milk (sometimes called skim, fat-free, or low-fat milk; includes white and chocolate)?  *Beverage Screener Questionnaire* | Never or less than 1 per week;  1 per week  2-4 per week  5-6 per week  1 per day  2-3 per day 4+ per day | 1 = 1-4+ per day/5-6 per week 0 = Never or less than 1 per week/1-4 per week |
| When you have milk, how often do you choose low-fat milk (skim or 1%)? *Cooking Matters (adult, teen)* | Never  Rarely  Sometimes  Often  Always  Does not apply | 1 = Always/Often 0 = Sometimes/Rarely/Never/Does not apply |
| Yesterday, how many times did you drink nonfat or 1% low-fat milk? Include low-fat chocolate or flavored milk and low-fat milk on cereal.  *EFNEP* | None 1 time  2 times  3 times  4 or more times | 1 = 1-4 or more times 0 = None |
| How often do you drink low-fat milk (skim or 1%)?  *WebNEERS* | N/A  Never  Seldom  Sometimes  Most times  Always | 1 = Always/most times 0 = Sometimes/Seldom/Never/NA |
| I (and the people in my home) drink low-fat or fat-free milk (included with cereal,) milk products (e.g. yogurt or cheese) or fortified soy milk)  *Recipe for Success Survey* | Never  Seldom  Sometimes  Most times  Always | 1 = Always/Most times 0 = Sometimes/Seldom/Never |
| How often do you drink low-fat or fat-free milk?  *North Carolina State University, Steps for Health, POC (youth)* | Always  Sometimes  Never | 1 = Always 0 = Sometimes/Never |
| What milk do you drink?  *Alabama Department of Public Health* | Whole milk  Low fat  None | 1 = Low fat 0 = Whole milk/None |
| I drink low-fat (1%) or non-fat milk (including soy milk or almond milk).  *Youth Behavior Survey, Grades 3-5 and Grades 6-12*  *Adult Behavior Survey* | 0 days  1-3 days  4-6 days  7 days | 1 = 4-7 days 0 = 3-0 days |
| What milk do you drink at home?  *Alabama Department of Public Health (youth)* | Whole milk  Low fat (school milk)  None | 1 = Low fat (school milk) 0 = Whole milk/None |
| How often do you drink low-fat or fat-free milk products?  *WEALTH (adults)* | Always  Most of the time  Sometimes  Seldom  Never | 1 = Always/Most of the time 0 = Sometimes/Seldom/Never |
| When you have milk, how often do you choose low-fat milk (skim or 1%)?  *Live Well Alabama* | Never  Rarely  Sometimes  Often  Always | 1 = Always/Often 0 = Sometimes/Rarely/Never |
| I drink low-fat (1%) or non-fat milk (including soy milk or almond milk).  *HomeStyles*  *MyPlate for my Family* | 0 days  1-3 days  4-6 days  7 days | 1 = 4-7 days 0 = 3-0 days |

Supplemental Table 6. Instrument recoding guidelines for cups of fruit (MT1l)

| **Question**  ***Source(s)*** | **Response Categories** | **Recode of Response Categories** |
| --- | --- | --- |
| I (and the people in my home) consume _____ cups of fruits per day (per person)  *Recipe for Success Survey* | Zero One Two Three Four | Mean |
| In the past week, how many cups of fruit did you eat each day? This includes fresh, frozen, canned and 100% juice.  *Live Well Alabama* | None  1 cup  2 cups  3 cups  4+ cups | Mean |
| On average, how many total cups (use your fist as a measure of a cup) of fruit do you eat each day? Count all that you eat whether in a combination dish or by itself.  *Steps for Health, North Carolina State University* | 0  1/2  1  1 1/2  2  2 1/2  3  3 1/2  4 or more | Mean |
| How many total cups of FRUITS do you typically eat in a day?  *University of South Carolina* | 1 cup  2 cups  3 or more cups  I don't usually eat fruit  I don't know | Mean of response categories with recode 4 to 0 |
| *24 Hour Recall* | Cups of fruits | Mean |

Supplemental Table 7. Instrument recoding guidelines for cups of vegetables (MT1m)

| **Question**  ***Source(s)*** | **Response Categories** | **Recode of Response Categories** |
| --- | --- | --- |
| I (and the people in my home) consume _____ cups of vegetables per day.  *Recipe for Success Survey* | Zero One Two Three Four | Mean |
| In the past week, how many cups of vegetables did you eat each day? This includes fresh, frozen, canned and 100% juice.  *Live Well Alabama* | None  1 cup  2 cups  3 cups  4+ cups | Mean |
| On average, how many total cups (use your fist as a measure of a cup) of vegetables do you eat each day? Count all that you eat whether in a combination dish or by itself.  *Steps for Health, North Carolina State University* | 0  1/2  1  1 1/2  2  2 1/2  3  3 1/2  4 or more | Mean |
| How many VEGETABLES do you typically eat in a day?  *University of South Carolina* | 1 Cup  2 Cups  3 or more cups  I don't usually eat vegetables  I don't know | Mean of response categories with recode 4 to 0 |
| *24 Hour Recall* | Cups of vegetables | Mean |

Supplemental Table 8. Instrument recoding guidelines for choose healthy foods for my family on a budget (MT2a)

| **Question**  ***Source(s)*** | **Response Categories** | **Recode of Response Categories** |
| --- | --- | --- |
| I choose healthy foods for my family.  *Body Quest Parent* | No  Sometimes  Often  Very Often  Almost Always | 1 = Almost Always/Very Often/Often 0 = Sometimes/No |
| How confident are you that you can buy healthy foods for your family on a budget?  *Cooking Matters (adults)* | Not at all confident  Not very confident  Neutral  Somewhat confident  Very confident  Does not apply | 1 = Very confident 0 = Somewhat confident/Neutral/Not very confident/Not at all confident/Does not apply |
| When deciding what to feed your family, how often do you think about healthy food choices?  *WebNEERS* | Never Rarely  Sometimes  Often  Always | 1 = Always/Often 0 = Sometimes/Rarely/Never |
| I choose healthy food for my family on a budget.  *EFNEP Checklist* | Never  Seldom  Sometimes  Most times  Always | 1 = Always/Most times 0 = Sometimes/Seldom/Never |
| I can choose and buy healthy foods on a tight budget.  *Alabama Department of Public Health (adults)* | True  False | 1 = True 0 = False |
| *Do you think about healthy food choices when deciding what to feed your family?*  *Food Stamp Food Behavior Checklist* | Do not do  Seldom  Sometimes  Most of the time  Almost always | 1 = Almost always/Most of the time 0 = Sometimes/Seldom/Do not do |
| How often do you choose healthy food for your family on a budget?  *WEALTH* | Always  Most of the time  Sometimes  Seldom  Never | 1 = Always/Most of the time 0 = Sometimes/Seldom/Never |

Supplemental Table 9. Instrument recoding guidelines for read nutrition facts labels or ingredients lists (MT2b)

| **Question**  ***Source(s)*** | **Response Categories** | **Recode of Response Categories** |
| --- | --- | --- |
| I use this food label. (with image)  *Body Quest Parent* | No  Sometimes  Often  Very Often  Almost Always | 1 = Almost Always/Very Often/Often 0 = Sometimes/No |
| How often do you use the “nutrition facts” on food labels?  *Cooking Matters (adults)* | Never  Rarely  Sometimes  Often  Always  Does not apply | 1 = Always/Often 0 = Sometimes/Rarely/Never |
| How often do you use the "Nutrition Facts" on the food label to make food choices?  *EFNEP* | Don't do  Seldom  Sometimes  Most of the time  Almost always | 1 = Almost always/Most of the time 0 = Sometimes/Seldom/Don't do |
| How often do you use the “Nutrition Facts” on the food label to make food choices?  *WebNEERS* | Never  Rarely  Sometimes  Often  Always  Does not apply | 1 = Always/Often 0 = Sometimes/Rarely/Never |
| I read nutrition fact labels or nutrition ingredient list.  *EFNEP Checklist* | Never Rarely  Sometimes  Often  Always | 1 = Always/Often 0 = Sometimes/Rarely/Never |
| How often do you use the "Nutrition Facts" on food labels to make food choices?  *HealthMPowers* | Never  Rarely  Sometimes  Often  Always | 1 = Always/Often 0 = Sometimes/Rarely/Never |
| Do you use this label when food shopping?  *Food Stamp Program Food Behavior Checklist* | No.  Yes sometimes.  Yes often.  Yes always. | 1 = Yes, always/Yes, often 0 = Yes, sometimes/No |
| How do you ... Use "nutrition facts" on food labels?  *University of South Carolina* | Never  Rarely  Sometimes  Often  Always  Does not apply | 1 = Always/Often 0 = Sometimes/Rarely/Never/Does not apply |
| How often do you use the "Nutrition Facts" on the food label to make food choices?  *Live Well Alabama* | Never  Rarely  Sometimes  Often  Always | 1 = Always/Often 0 = Sometimes/Rarely/Never |
| How often do you read nutrition fact labels and nutrition ingredient lists?  *WEALTH (adults)* | Always  Most of the time  Sometimes  Seldom  Never | 1 = Always/Most of the time 0 = Sometimes/Seldom/Never |
| I read nutrition facts labels and/or ingredient lists.  *Alabama Department of Public Health (adults)* | True  False | 1 = True 0 = False |
| Do you use the “Nutrition Facts” label to compare foods when shopping?  *University of Georgia* | Do not do  Seldom  Sometimes  Most of the time  Almost always | 1 = Almost always/Most of the time 0 = Sometimes/Seldom/Do not do |

Supplemental Table 10. Instrument recoding guidelines for not run out of food before month’s end (MT2g)

| **Question**  ***Source(s)*** | **Response Categories** | **Recode of Response Categories** |
| --- | --- | --- |
| I run out of food before the end of the month.  *Body Quest Parent* | No  Sometimes  Often  Very often  Almost always | 1 = No 0 = Sometimes/Often/Very often/Almost always |
| How often do you worry that your food might run out before you get money to buy more?  *Cooking Matters (adults)* | Never  Rarely  Some-times  Often  Always  Does not apply | 1 = Never/Rarely 0 = Sometimes/Often/Always/Does not apply |
| How often do you run out of food before the end of the month?  *EFNEP* | Don't do Seldom Sometimes Most of the time Almost always | 1 = Don't do/Seldom 0 = Sometimes/Most of the time/Almost always |
| How often do you run out of food before the end of the month?  How often do you run out of food before the month's end?  *EFNEP* | Never to Always (1-5) | 1 = 1 or 2 0 = 3, 4, or 5 |
| I (and the people in my home) run out of food before the month’s end.  *EFNEP Checklist* | Never  Seldom  Sometimes  Most times  Always | 1 = Never/Seldom 0 = Sometimes/Most times/Always |
| How often do you ... Run out of money for more food?  *University of South Carolina* | Never  Rarely  Sometimes  Often  Always  Does not apply | 1 = Never/Rarely 0 = Sometimes/Often/Always/Does not apply |
| How often do you worry that your food might run out before you can buy more?  *Steps for Health, North Carolina State University* | Never  Rarely  Sometimes  Often  Always  Does not apply | 1 = Never/Rarely 0 = Sometimes/Often/Always/Does not apply |
| I run out of food before the end of the month.  *ESBA Checklist* | No  Sometimes  Often  Very often  Almost always | 1 = No 0 = Sometimes/Often/Very often/Almost always |
| Do you run out of food before the end of the month?  *University of Georgia* | Do not do  Seldom  Sometimes  Most of the time  Almost always | 1 = Do not do/Seldom 0 = Sometimes/Most of the time/Almost always |
| How often do you not run out of food before the end of the month?  *WEALTH (adults)* | Always  Most of the time  Sometimes  Seldom  Never | 1 = Most of the time/Always 0 = Sometimes/Seldom/Never |
| How often do you run out of food before the end of the month?  *Live Well Alabama* | Never  Rarely  Sometimes  Often  Always | 1 = Never/Rarely 0 = Sometimes/Often/Always |

Supplemental Table 11. Instrument recoding guidelines for compare prices before buying foods (MT2h)

| **Question**  ***Source(s)*** | **Response Categories** | **Recode of Response Categories** |
| --- | --- | --- |
| I compare prices.  *Body Quest Parent* | No  Sometimes  Often  Very often  Almost always | 1 = Often/Very often/Almost always 0 = No/Sometimes |
| How often do you compare prices before you buy food?  *Cooking Matters (adults)* | Never  Rarely  Sometimes  Often  Always  Does not apply | 1 = Often/Always 0 = Never/Rarely/Sometimes/Does not apply |
| How often do you compare prices before you buy food?  *EFNEP* | Don't do  Seldom  Sometimes  Most of the time  Almost always | 1 = Almost always/Most of the time 0 = Sometimes/Seldom/Don't do |
| How often do you compare prices before you buy food?  *WebNEERS* | Never to Always (1-5) | 1 = 4, 5 0 = 1, 2, 3 |
| I compare prices before buying food.  *EFNEP Checklist* | Never  Seldom  Sometimes  Most Times  Always | 1 = Always/Most times 0 = Sometimes/Seldom/Never |
| Do you compare prices before you buy food?  *University of Georgia* | Do not do  Seldom  Sometimes  Most of the time  Almost always | 1 = Almost always/Most of the time 0 = Sometimes/Seldom/Do not do |
| How often do you ... compare prices?  *University of South Carolina* | Never  Rarely  Sometimes  Often  Always  Does not apply | 1 = Always/Often 0 = Sometimes/Never/Rarely/Does not apply |
| How often do you compare prices before you buy food?  *Steps for Health, North Carolina State University* | Never  Seldom  Sometimes  Most of the time  Almost always | 1 = Almost always/Most of the time 0 = Sometimes/Seldom/Never |
| I compare the prices of foods before buying them.  *Alabama Department of Public Health (adults)* | Every time I shop  Only sometimes when I shop  Never | 1 = Every time I shop 0 = Only sometimes when I shop/Never |
| I compare prices.  *ESBA Checklist* | No  Sometimes  Often  Very often  Almost always | 1 = Almost always/Very often/Often 0 = Sometimes/No |
| How often do you compare prices before buying foods?  *WEALTH (adults)* | Never  Seldom  Sometimes  Most of the time  Always | 1 = Always/Most of the time  0 = Sometimes/Seldom/Never |
| How often do you compare prices before you buy food?  *Live Well Alabama* | Never  Rarely  Sometimes  Often  Always | 1 = Always/Often 0 = Sometimes/Rarely/Never |

Supplemental Table 12. Instrument recoding guidelines for identify foods on sale or use coupons to save money (MT2i)

| **Question**  ***Source(s)*** | **Response Categories** | **Recode of Response Categories** |
| --- | --- | --- |
| I change meals to include some ingredients that are more "budget friendly," like on sale.  *Body Quest Parent* | No  Sometimes  Often  Very often  Almost always | 1 = Almost always/Very often/Often 0 = Sometimes/No |
| How often do you adjust meals to include specific ingredients that are more “budget-friendly,” like on sale or in your refrigerator or pantry?  *Cooking Matters (adults)* | Never  Rarely  Sometimes  Often  Always  Does not apply | 1 = Always/Often 0 = Sometimes/Rarely/Never/Rarely/Does not apply |
| How often do you use a coupon to identify foods on sale?  *WebNEERS* | Never to Always (1-5) | 1 = 5, 4 0 = 1, 2, 3 |
| I identify foods on sale or coupons to save money.  *EFNEP Checklist* | Never  Seldom  Sometimes Most Times  Always | 1 = Always/Most times 0 = Sometimes/Seldom/Never |
| How often do you adjust meals to include specific ingredients that are more "budget friendly," like on sale?  *HealthMPowers* | Never  Rarely  Sometimes  Often  Always  Does not apply | 1 = Always/Often 0 = Sometimes/Rarely/Never/Does not apply |
| How often do you ... look for foods on sale?  *University of South Carolina* | Never  Rarely  Sometimes  Often  Always  Does not Apply | 1 = Always/Often 0 = Sometimes/Rarely/Never/Does not apply |
| How often do you adjust meals to include some ingredients that are more "budget friendly" like on sale?  *Steps for Health, North Carolina State University* | Never  Seldom  Sometimes  Most of the time  Almost always | 1 = Almost always/Most of the time 0 = Sometimes/Seldom/Never |
| How often do you identify foods on sale or use coupons to save money?  *WEALTH (adults)* | Always  Most of the time  Sometimes  Seldom  Never | 1 = Always/Most of the time 0 = Sometimes/Seldom/Never |
| I look for items on sale at the grocery store.  *Alabama Department of Public Health* | Every time I shop  Only sometimes when I shop  Never | 1 = Every time I shop 0 = Only sometimes when I shop/Never |

Supplemental Table 13. Instrument recoding guidelines for shop with a list (MT2j)

| **Question**  ***Source(s)*** | **Response Categories** | **Recode of Response Categories** |
| --- | --- | --- |
| I shop with a list.  *Body Quest Parent* | No  Sometimes  Often  Very often  Almost always | 1 = Almost always/Very often/Often 0 = Sometimes/No |
| How often do you use a grocery list when you go grocery shopping?  *Cooking Matters (adults)* | Never  Rarely  Sometimes  Often  Always  Does not apply | 1 = Always/Often 0 = Sometimes/Rarely/Never/Does not apply |
| How often do you shop with a grocery list?  *EFNEP* | Don't do  Seldom  Sometimes  Most of the time  Almost always | 1 = Almost always/Most of the time 0 = Sometimes/Seldom/Don't do |
| How often do you shop with a grocery list?  *WebNEERS* | Never to Always (1-5) | 1 = 5, 4 0 = 1, 2, 3 |
| I shop with a list.  *EFNEP Checklist* | Never  Seldom  Sometimes  Most times  Always | 1 = Always/Most times 0 = Sometimes/Seldom/Never |
| How often do you ... use a shopping list?  *University of South Carolina* | Never  Rarely  Sometimes  Often  Always  Does not apply | 1 = Always/Often 0 = Sometimes/Rarely/Never |
| How often do you shop with a grocery list?  *Live Well Alabama* | Never  Rarely  Sometimes  Often  Always | 1 = Always/Often 0 = Sometimes/Rarely/Never |
| How often do you shop with a grocery list?  *Steps for Health, North Carolina State University* | Never  Seldom  Sometimes  Most of the time  Almost always | 1 = Almost always/Most of the time 0 = Sometimes/Seldom/Never |
| How often do you shop with a grocery list?  *WEALTH (adults)* | Never  Seldom  Sometimes  Most of the time  Always | 1 = Always/Most of the time 0 = Sometimes/Seldom/Never |
| I always take a list when shopping at the grocery store.  *Alabama Department of Public Health (adults)* | True  False | 1 = True 0 = False |
| I shop with a list.  *ESBA Checklist* | No  Sometimes  Often  Very often  Almost always | 1 = Almost always/Very often/Often 0 = Sometimes/No |
| Do you shop with a grocery list?  *University of Georgia* | Do not do Seldom Sometimes Most of the time Almost always | 1 = Almost always/Most of the time 0 = Sometimes/Seldom/Do not do |

Supplemental Table 14. Categories Used to Report Policy, Systems, Environmental, and Promotional Changes (MT5)^a^

| **POLICY CHANGE (MT5b)** | **SYSTEMS CHANGE (MT5c)** | **ENVIRONMENTAL CHANGE (MT5d)** | **PROMOTIONAL CHANGE (MT5e)** |
| --- | --- | --- | --- |
| 1. Improvements in hours of operations/time allotted for meals or food service | 1. Improved child feeding practices (e.g. served family style, adults role model healthy behaviors, etc.) | 1. Improvements in layout or display of food (Smarter Lunchrooms, worksite cafeterias) | 1. Point-of-purchase/distribution prompts |
| 2. Policies for working mothers | 2. Implemented guidelines for healthier competitive foods options | 2. Edible gardens (establish, reinvigorate or maintain food gardens | 2. Menu labeling/calorie/fat/ sodium/added sugar counts |
| 3. Rules on foods served in meetings or in classrooms | 3. Implemented a system to involve youth in food service decision-making | 3. Lactation supports or dedicated lactation space | 3. Vending machine labeling |
| 4. Standards for healthier food policy in other setting | 4. Removing sugar-sweetened beverages from children’s menus | 4. Healthier vending machine initiatives (e.g., access to healthier foods and beverages) | 4. Increased awareness of changes reported by target audience |
| 5. School wellness or child care wellness policy implemented | 5. Enhanced training on menu design and healthy cooking techniques | 5. Improved appeal, layout, or display of healthy snack foods | 5. Used posters/visual displays, taste testing, live demonstrations, audiovisuals, celebrities, etc. |
| 6. Established or improved a monitoring and reporting system for school or child care wellness policies | 6. Use of standardized, healthful recipes | 6. Improved appeal, layout or display of healthy competitive foods | 6. Implemented or enhanced limits on marketing/promotion of less healthy options |
| 7. Established or improved a nutrition policy | 7. Change in/improved menus (variety, quality, offering lighter fares) | 7. Eliminated or reduced amount of competitive foods | 7. School meal foods promoted outside of meal times |
| 8. Improved hours of operation to improve access/convenience | 8. Implemented, improved, or expanded healthy fundraisers | 8. Established or improved salad bar | 8. Meal service staff encourages healthy selections |
| 9. Took policy steps to encourage new food distribution sites - pantries, food banks | 9. Improvements in free water access, taste, quality, smell, or temperature | 9. Decreased shelf space/amount/variety of unhealthy options | 9. Outreach and promotion conducted to increase awareness and access to the site(s) (pantries, farmer's markets, new stores) |
| 10. Took policy steps to encourage new healthy retail outlets | 10. Restrictions on use of food as rewards or during celebrations | 10. Improved quality of healthy options | 10. Took promotional steps to encourage new food distribution sites |
|  | 11. Implemented guidelines for healthier snack options | 11. Created or enhanced healthy check out area | 11. Took promotional steps to encourage new healthy retail outlets |
|  | 12. Improved or increased healthy beverage options | 12. Improved appeal, layout, or display of foods to encourage healthy and discourage unhealthy selections |  |
|  | 13. Implemented a system for youth, parent, and/or client leadership or involvement in decision-making | 13. Increased shelf space/amount/variety of healthy options |  |
|  | 14. Implemented guidelines for foods offered during events, celebrations, education programs, etc., not at schools/day care | 14. Established a new food bank, pantry, or distribution site |  |
|  | 15. Change in/improved vendor agreement towards healthier food(s) | 15. Established a new healthy retail outlet |  |
|  | 16. Change in/improved food purchasing/donation specifications towards healthier food(s) | 16. Improved or expanded cafeteria/dining/serving areas or facilities - if allowable |  |
|  | 17. Prioritized farm-to-table/increase in fresh or local produce | 17. Improved facilities or equipment to accommodate healthier options or make them more convenient/appealing/access-ible - if allowable |  |
|  | 18. Expanded or improved transportation options to the site | 18. Improved or expanded kitchen/food preparation facilities - if allowable |  |
|  | 19. Began offering a federal food program (CACFP, TEFAP, summer meals, etc.) |  |  |
|  | 20. Implemented novel distribution systems to reach high-risk population (e.g. home delivery of the elderly, farmers’ markets) |  |  |
|  | 21. Began acceptance of SNAP/EBT/WIC |  |  |
|  | 22. Farmers market established in food banks |  |  |
|  | 23. Fresh produce made accessible in food pantries |  |  |
|  | 24. Implemented nutrition standards for foods accepted and distributed in food pantries and food banks |  |  |
|  | 25. Collected or accepted donations of excess wholesome food to distribute to clients |  |  |
|  | 26. Offered on-site enrollment in federal food programs - if allowable |  |  |
|  | 27. Improved enrollment procedures to increase NSLBP meal participation including universal breakfast/lunch - if allowable |  |  |
|  | 28. Implemented price manipulation/coupons/discounts to encourage healthy choices - if allowable |  |  |

^a^ As listed in the SNAP-Ed Evaluation Framework.
